# Pericapsular nerve group block for osteoarthritis-related chronic hip joint pain: a case report

**DOI:** 10.1186/s40981-023-00673-1

**Published:** 2023-11-14

**Authors:** Reiko Sato, Rina Kato, MinHye So, Takeshi Sugiura, Kazuya Sobue

**Affiliations:** Department of Anesthesiology and Intensive Care Medicine, Nagoya City University Graduate School of Medical Sciences, Kawasumi, Mizuho-Cho, Mizuho-Ku, Nagoya, 467-8602 Japan

**Keywords:** Pericapsular nerve group (PENG) block, Hip osteoarthritis, Chronic hip joint pain

## Abstract

**Background:**

Pericapsular nerve group (PENG) block has shown effectiveness for acute hip pain associated with fractures and surgery. Herein, PENG block was performed for osteoarthritis (OA)-related chronic hip joint pain.

**Case presentation:**

A 65-year-old woman presented left hip pain. She had bilateral hip osteoarthritis that improved with medications; however, a fall resulted in left hip pain. She experienced severe pain on movements, which required walking aids. To alleviate the hip pain, a PENG block was performed under ultrasound guidance. Transient muscle weakness occurred in 2 of 5 times. After 5 blocks, she regained the ability to walk without assistive devices. Pain did not recur even after 6 months.

**Conclusions:**

Repeated PENG blocks of short-acting local anesthetics alone could be an effective pain management technique for chronic hip pain. For safety, the appropriate injection site and local anesthetic dosage must be carefully considered.

## Background

Recently, ultrasound-guided pericapsular nerve group (PENG) block has attracted significant attention as a new analgesic technique for hip pain. Its efficacy has been reported in acute hip pain cases stemming from fractures or surgeries [1]. A single PENG block using a long-acting local anesthetic with steroid provides effective pain relief in hip osteoarthritis (OA) patients for up to 2 months; however, its long-term effectiveness was limited [2]. This paper focused on the repetitive application of PENG blocks with lidocaine, which led to sustained pain reduction and overall mobility in a case of OA-related chronic hip pain.

## Case presentation

The patient was a 65-year-old woman (height 159 cm, weight 63 kg, body mass index 25.2). She sought care from our orthopedic surgery department, where was treated for lumbar spinal stenosis with oral tramadol hydrochloride (75 mg/day). In addition, postherpetic neuralgia in the right upper extremity was treated using the right stellate ganglion xenon irradiation and oral pregabalin (150 mg/day). Six months ago, she experienced right hip pain after a long walk and was diagnosed with bilateral hip joint OA. Following 2 months of celecoxib therapy, the pain improved, prompting her to discontinue the medication and undergo subsequent follow-ups. Three months before the presentation, a fall resulted in left hip pain. A post-fall X-ray examination did not find fractures, other anomalies, or observable joint alterations. Although she resumed taking celecoxib, the pain remained. The main painful site was the left groin area, with maximum pain intensity of 8 on the numerical rating scale (NRS) while walking. This persistent pain prompted her to use a cane or other ambulatory aids.

**Fig. 1:**
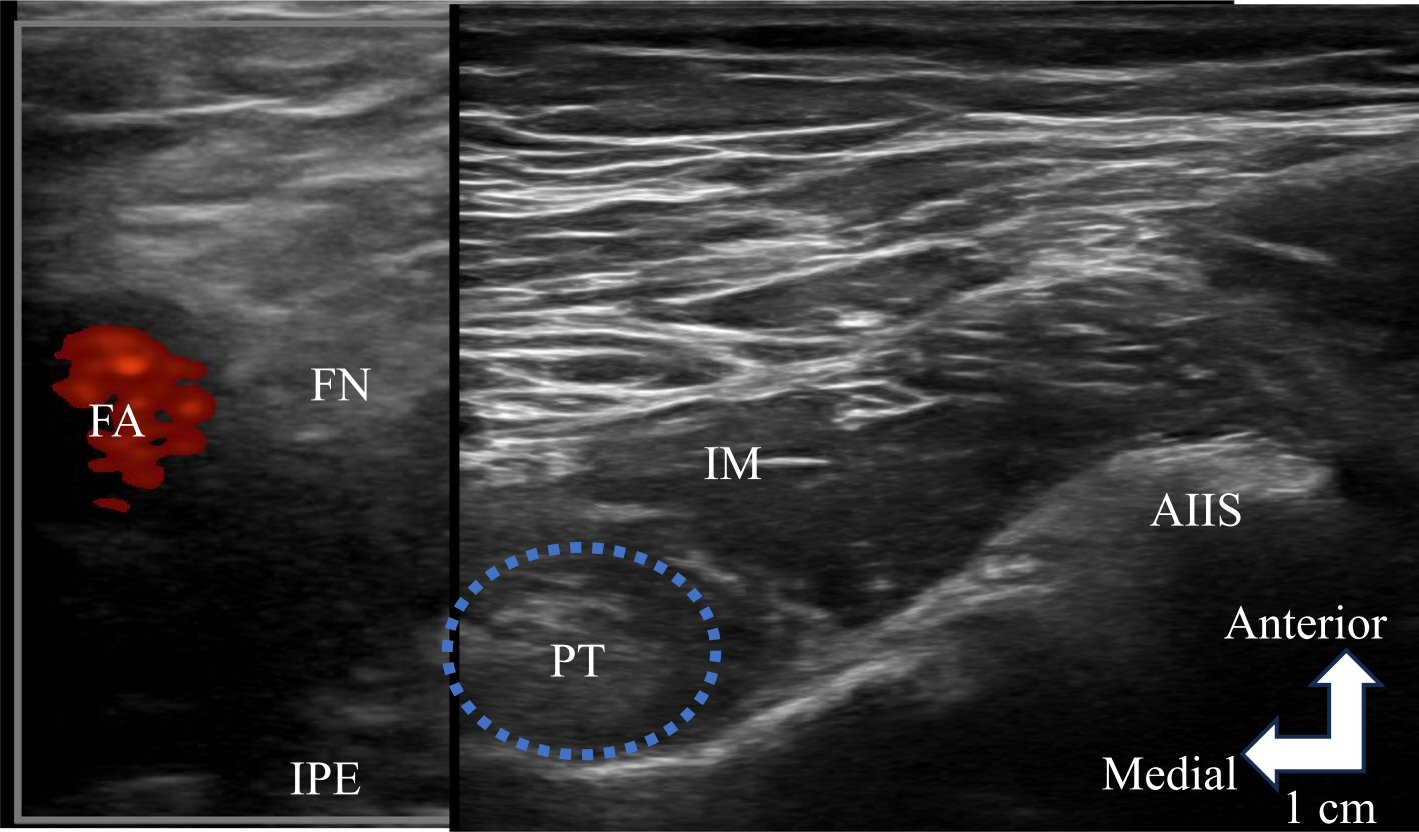
Relevant sonoanatomy view for the PENG block. The needle was inserted using a lateral in-plane approach to direct the tip within the myofascial plane, positioned between the psoas tendon anteriorly and the ilium posteriorly. AIIS, anterior inferior iliac spine; FA, femoral artery; FN, femoral nerve; IM, iliac muscle; IPE, iliopubic eminence; PT, psoas tendon

**Fig. 2:**
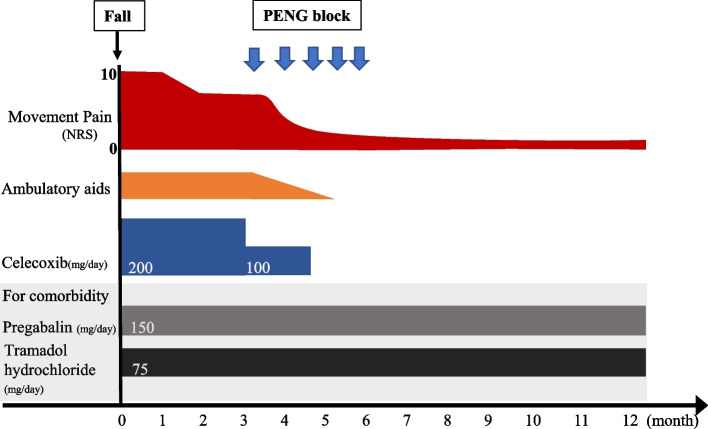
Our patient’s post-fall clinical course. Three months after falling, the patient was taking celecoxib but continued to experience pain. A series of PENG blocks reduced NRS from 8 to 1–2. After the third injection, celecoxib was no longer needed; she discontinued cane use after the fourth injection

To precisely determine the source of pain and alleviate severe pain, a left PENG block using 15 mL of 0.5% lidocaine was administered under ultrasound guidance. During the procedure, the patient was positioned supine. Briefly, an ultrasound probe was placed transversely, and the anterior inferior iliac spine, iliopubic eminence, and psoas tendon were identified (Fig. 1). Subsequently, a needle was inserted via a lateral in-plane approach to guide the tip within the myofascial plane, positioned between the psoas tendon anteriorly and the ilium posteriorly. Thereafter, 15 mL of 0.5% lidocaine was gradually injected. No complications or side effects associated with PENG block were observed, except for transient quadriceps weakness. Following the initial block, pain improved during ambulation. Following five subsequent block applications at intervals of 2–4 weeks, the pain stabilized at NRS 1–2, facilitating independent walking. To date, pain has not recurred even after 6 months (Fig. 2).

## Discussion

**Fig. 3:**
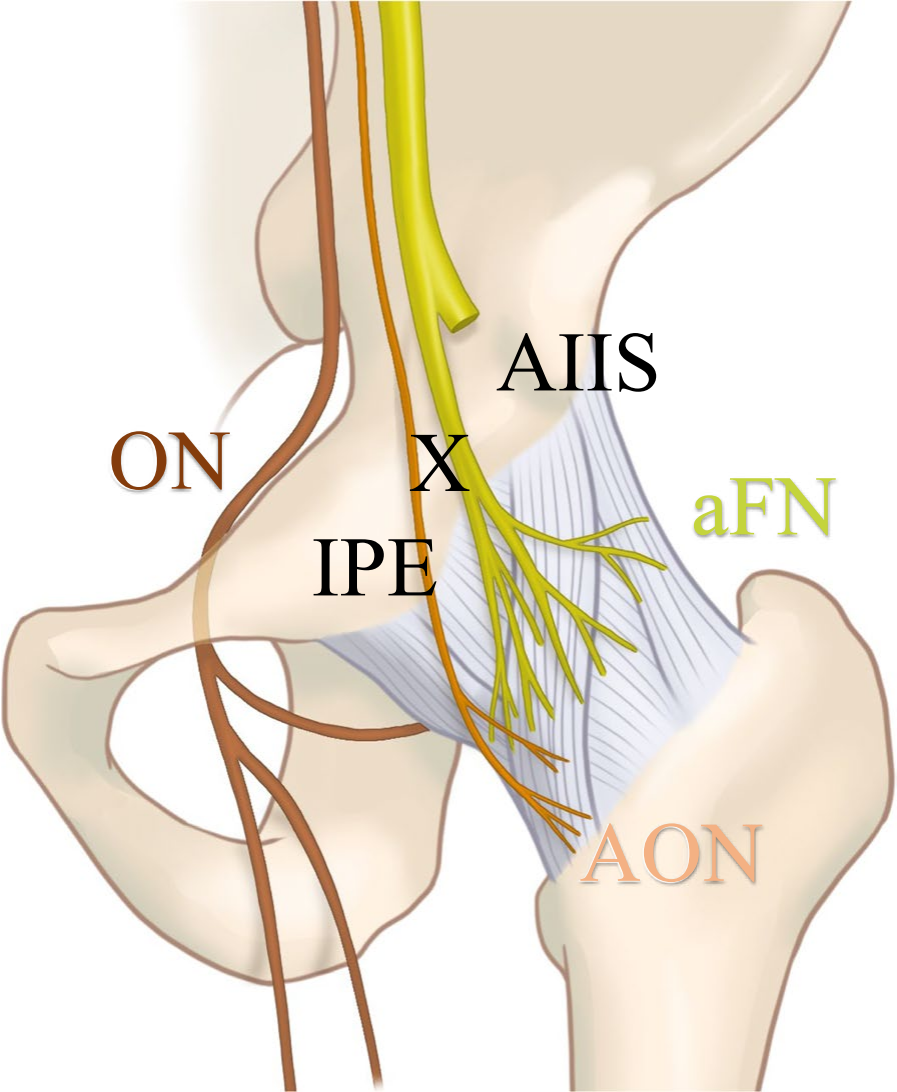
Branches of the lumbar plexus that innervate anterior hip capsule. The obturator nerve exhibits a comparatively greater distance from the intended injection site (X). The femoral and accessory obturator nerves pass between the anterior inferior iliac spine (AIIS) and the iliopubic eminence (IPE). aFN, articular branch of the femoral nerve; AIIS, anterior inferior iliac spine, AON, accessory obturator nerve; ON, obturator nerve; IPE, iliopubic eminence

The anterior hip joint is innervated by the femoral, obturator, and accessory obturator nerves and is therefore a focal point for hip analgesia (Fig. 3) [3]. PENG block, introduced in 2018, targets the sensory branches, including the femoral nerve articular branch and accessory obturator nerve [1]. Studies have highlighted the application of PENG block for fracture- or hip surgery-related acute pain [1, 4, 5]. In OA-related chronic hip joint pain, a single PENG block with a long-acting local anesthetic and steroid was initially as effective as conventional intra-articular steroid injections over the short term; however, its effect diminished over the ensuing weeks [2]. Presently, no evidence demonstrates the longer-term efficacy of utilizing short-acting local anesthetics repeat.

In our case, repeated PENG blocks maintained an analgesic effect. Unlike a single-shot PENG block, which shows reduced analgesic effectiveness after 4 weeks [2], repeated treatments at 2–4 week intervals provided longer-lasting relief. While such repeat blocks may increase medical costs and complications, they are a convenient and safe alternative to intra-articular injections and avoid the risk of septic arthritis. Therefore, repeated PENG blocks could serve as a viable therapy option.

PENG block emerges as a potentially valuable diagnostic and therapeutic tool for patients like ours, in whom severe hip pain persists despite relatively minor joint deformity. Plain X-ray findings do not reliably correlate with pain symptoms in OA [6]. Here, post-fall imaging showed no significant changes, suggesting that the pain likely stems from inflammation resulting from minor soft tissue damage. Inflammatory conditions cause the sensitization at the spinal dorsal horn cell. Speculatively, blocking nerve inputs with a short-acting local anesthetic can reduce hypersensitivity and offer prolonged pain relief for 6 months. Additional studies are required to determine the suitability of PENG block for chronic hip pain.

We initially perceived that using a PENG block would be less likely to cause muscle weakness [1] because it blocks the articular branches (Fig. 3). However, quadriceps muscle weakness arises in 25% of the patients after PENG block [4]. Therefore, we suggested that a high injection pressure or large drug volumes may precipitate muscle weakness [7]. The injection site in the PENG block aligns with the synovial capsule. Synovial capsule rupture because of injection can result in drug dispersion across the fascial surface and between muscles reaching the primary trunk of the femoral or obturator nerve, inducing anesthesia [7]. In the original report [1], 20 mL was employed for the PENG block; even with 10 mL, dispersion into the anterior hip joint capsule was observed [8]. Although 15 mL may be considered excessive, it is possible that a high-pressure or inappropriate injection site may have existed. We chose a short-acting local anesthetic without a steroid and observed that the muscle weakness disappeared after 1 h, only minimally extending the outpatient visit duration. For safety in an outpatient context, appropriate local anesthetic dosage and injection sites must be meticulously considered.

In managing OA-related chronic hip pain, PENG block presents as a promising option for conservative therapy. Ultrasound-guided PENG block offers simplicity within an outpatient clinical setting. However, the potential risk of inducing muscle weakness, emphasizing the need for meticulous deliberation over optimal dosage and administration sites, must be analyzed.

## Data Availability

Not applicable.

## Abbreviations

NRS: Numerical rating scale
PENG: Pericapsular nerve group
OA: Osteoarthritis
